# Integrative Perinatal Management Enhanced the Advantage of Prenatal Diagnosis on Critical Pulmonary Valve Stenosis: An Observational Preliminary Study

**DOI:** 10.3389/fped.2020.572238

**Published:** 2020-12-21

**Authors:** Jiawen Li, Gang Li, Xiaoqing Shi, Chuan Wang, Hongyu Duan, Kaiyu Zhou, Yimin Hua, Yifei Li

**Affiliations:** ^1^Department of Pediatrics, West China Second University Hospital, Sichuan University, Chengdu, China; ^2^Ministry of Education Key Laboratory of Women and Children's Diseases and Birth Defects, West China Second University Hospital, Sichuan University, Chengdu, China; ^3^Department of Pediatrics, The Affiliated Hospital of Southwest Medical University, Luzhou, China

**Keywords:** critical pulmonary stenosis, PBPV, integrative perinatal management, prognosis, right ventricular function restore

## Abstract

**Background:** Percutaneous balloon valvuloplasty (PBPV) is recommended as a first-choice treatment for critical pulmonary stenosis (CPS). A concept of perinatal integrative management has been developed. Unfortunately, the evidence on the advantage of integrative management for CPS during the perinatal period is absent.

**Methods:** Single-center, observational, preliminary research has been developed, and three groups have been enrolled. There were 42 children with CPS enrolled for this study between January 2014 and December 2017 in our center, and their follow-up duration is at least 1 year. Three groups were set up: the integrative perinatal management group (group I), who received prenatal diagnosis with perinatal management to maintain circulation and an optimized PBPV procedure; the prenatal diagnosis group (group PR), who received a diagnosis of pulmonary stenosis before birth without any monitoring and perinatal management; and the postnatal diagnosis group (group PO), who received the CPS diagnosis after birth.

**Result:** There were 13 patients enrolled in group I, 11 babies enrolled in group PR, and 18 cases included in group PO. Integrative management helped to put the timing of PBPV in advance. The age for PBPV in group I was 9.38 ± 5.58 days, and groups PR and PO were 24.54 ± 4.87 and 49.11 ± 9.50 days, respectively. The average peak transvalvular gradient (PGs) of the perinatal management group (group I) and prenatal diagnosis group (group PR) remained at a stable level. However, the average PGs of group PO were progressively elevated during follow-up. Moreover, the follow-up data from group I revealed an advantage in RV development and functional restoration. There was no difference among the three groups in the ratio of reintervention and postoperative moderate pulmonary regurgitation during 1-year follow-up (*p* >0.05).

**Conclusion:** Prenatal diagnosis helps to improve the outcomes of PBPV. Moreover, perinatal integrative medical management enhances the advantage of prenatal diagnosis. However, this research is still a small-size cohort study, and the limited population number and follow-up duration were the major limitations to expand the conclusions.

## Introduction

Critical pulmonary stenosis (CPS) is a type of ductal-dependent disease, along with pulmonary atresia with intact ventricular septum (PAIVS), which account for 3% of all congenital heart diseases (CHD) (1). Patients predominantly present with severe cyanosis and right ventricular (RV) dysfunction, which might lead to right ventricular hypoplasia during fetal and neonatal periods (2). Besides this, the natural mortality of CPS is very high if patients fail to receive timely treatment after birth (3). Percutaneous balloon pulmonary valvuloplasty (PBPV) is considered the most common treatment choice for CPS, which is predicted with biventricular heart management, although, for some single-ventricle predicted cases, surgical treatment, includes the Fontan, Glenn procedure (4, 5). PBPV performed in the early life of neonates can be conducted to alleviate hypoxemia, reduce high pressure on the RV, help to restore cardiac function, and save lives (6). Ronai et al. reported that fetal cardiac intervention is likely to provide better outcomes in some cases because it would be difficult to achieve acceptable ventricular function with extremely poor right heart hypoplasia (7). Other studies have confirmed the advantages of fetal PBPV in accelerating RV and pulmonary artery (PA) development (8–10). Theoretically, the earlier pulmonary valvuloplasty is conducted in CPS patients, the better the benefits from shortening the duration of hypoxemia exposure and more capabilities for RV function restoration to be achieved (11).

Therefore, prenatal diagnosis is important in guiding the optimal time for PBPV (12). Nowadays, a new concept of perinatal integrative management has been developed for some severe diseases that should be treated within a specific time window postnatally, and this is realized by collaboration among the pediatric cardiologist, obstetrician, ultrasound physician, neonatologist, and anesthesiologist (13). The integrative perinatal management procedure includes dynamic monitoring of peak transvalvular gradient (PG) by prenatal echocardiography, swift transfer from the delivery room to the neonatal intensive care unit, advanced life support, and finally making a clinical decision on PBPV within a specific time window. Therefore, it is considered that perinatal integrative management could provide a distinctive advantage in improving long-term prognosis and the quality of life for such patients and enhance the benefits from accurate prenatal diagnosis by adequate preoperative preparation and timely intervention.

Unfortunately, evidence on the advantages of integrative management for CPS during the perinatal period is lacking. Herein, we present our preliminary data on whether an integrative management strategy would influence the prognosis of CPS in the perinatal period.

## Materials and Methods

### Subjects

In this prospective, preliminary, and observational study, from January 2014 to December 2017, we recruited a cohort with CPS that presented with significant pressure differences across the affected area (>90 mmHg) based on echocardiography measurement or any evidence on RV and/or PA hypoplasia, and fetal echocardiography demonstrated a bidirectional shunt between the right and left atria. Z values of the tricuspid valve (TV) of all neonatal patients were more than −2.0 so that patients could predict biventricular circulation heart function. There were a total of 42 cases enrolled in this study. The parents of all included babies provided written consent for participating in the study carried out at the West China Second University Hospital, Sichuan University, China. Participant information can be retrieved by the individual registry number in our hospital. This research study was approved by the ethics committee of our hospital (2014-034). All enrolled patients were divided into three individual groups based on the following two parameters: (1) receipt of prenatal diagnosis and (2) receipt of integrative perinatal management.

The integrative perinatal management group (group I) included 13 babies who received prenatal diagnosis and were delivered under the administration of an integrative perinatal management team. The integrative management strategy team comprised a pediatric cardiologist, obstetrician, ultrasound physician, neonatologist, and anesthesiologist. This strategy included (1) a definitive prenatal diagnosis, including fetal cardiovascular structure, cardiac rhythm, and cardiac function; (2) active prenatal and perinatal monitoring, focusing on the development of fetal RV, progression of pulmonary valve stenosis, and cardiac function; (3) continuous intravenous infusion of alprostadil after birth at a dose of 5 ng/kg/min to maintain the opening of ductus arteriosus; (4) echocardiography used to evaluate the severity of pulmonary stenosis by PG between the RV and PA, morphology of the RV, circulation of the coronary artery, cardiac function, and cardiac rhythm in neonates; (5) essential preparation achieved for urgent PBPV within a limited time after birth; (6) no oxygen inhalation supplied before PBPV; (7) consideration of cesarean delivery to rescue fetal life and perform PBPV in a timely manner; (8) ACE inhibitors applied to the patients within 6 months after PBPV.

The prenatal diagnosis group (group PR) included 11 patients who had a clear pulmonary stenosis before birth but without perinatal monitoring of the development of CPS as well as lacking the integrative perinatal management. The postnatal diagnosis group (group PO) included 18 patients who only received the CPS diagnosis after birth. In all group PR and PO patients, echocardiography should be repeated to identify the CPS standard at the time of hospitalization, and emergency PBPV should be performed within 2 days after hospitalization. Intravenous infusion of alprostadil was not considered routine therapy in these two groups. Moreover, no oxygen inhalation was supplied before PBPV although ACE inhibitors should be administered to both groups after PBPV for half a year.

The exclusion criteria were as follows: (1) multiple pregnancy, (2) patients suffered CPS in combination with other types of cardiovascular malformation, (3) RV dependency on coronary circulation, (4) suspected cardiomyopathy, (5) any cases with pulmonary atresia, and (6) severe RV hypoplasia with a Z score of the TV that is lower than −2, which predicts a single ventricle outcome.

We prospectively recorded serial morphologic and physiological parameters before and after PBPV and during the 1-year follow-up in this analysis. The dynamic changes of Tei-index of RV and PG were monitored during the 1-year follow-up on PBPV to evaluate the efficacy of PBPV followed by different management strategies (I, PR, and PO groups).

### Measurements—Equipment

Echocardiographic assessments were performed using the Vivid 7 ultrasound system (GE Vingmed Ultrasound AS, Horten, Norway). Averages of echocardiographic indices measured from three cardiac cycles were obtained for statistical analysis (4).

### Measurements—Diagnostic Criteria

A comprehensive examination was conducted of the fetal and newborn hearts to assess the location and connection of the abdomen in combination with transverse and longitudinal views (3). CPS was diagnosed only if a string-like flow was present across the nearly atretic pulmonary valve during RV angiography and if a significant pressure difference was recorded across the affected area.

### Measurements

We assessed the RV and measured the pulmonary valve's valvular dimensions at the hinge points at end-systole (3). The RV Tei-index is defined as the sum of the RV isovolumic contraction and the isovolumic relaxation time divided by RV ejection time; therefore, it assesses RV function from both systolic and diastolic phases (14). The Z-score of the TV was evaluated before PBPV and at the end of the 1-year follow-up. A balloon-tipped, fluid-filled catheter was positioned in a main PA branch under multislice spiral CT guidance via the femoral approach. Pressure transducers were zeroed at the midaxillary level; simultaneous right atrial, RV, and PA pressures were recorded continuously. In the supine position, baseline pressures were recorded, and mixed venous blood was sampled for oximetry (15).

### Reproducibility

The PG and RV Tei index values represent the mean of three measurements made from the same image or a consecutive good quality Doppler strip. All fetal measurements and postnatal measurements were independently analyzed by two cardiologists (LY and HY), and any disagreement was resolved by a third cardiologist. Reproducibility of the two cardiologists was assessed using Bland–Altman analysis on 12 randomly selected cases (16).

### Intervention

Cardiac catheterization and PBPV were performed under general anesthesia. The right femoral artery and vein were punctured percutaneously, and 0.6 mg/kg heparin was injected intravenously for anticoagulation. RV and PA pressure were measured using a 4-5F RV catheter, and PG was calculated. Right ventriculography was performed to determine the type and severity of PS. To select the appropriate balloon diameter, the diameter of the pulmonary valve annulus was measured. A 4-5F multipurpose catheter or right coronary catheter was delivered from the right femoral vein to the distal end of the left lower PA via the RV. It was helpful to pass through the pulmonary valve orifice for patients with CPS, so a 0.014-inch coronary artery guide wire or a 0.025-inch super-slip guide wire was inserted into the PA through a multipurpose catheter or into the descending aorta through an arterial catheter. Next, the catheter and sheath were withdrawn, a balloon of suitable size (balloon/annulus ratio: 1:1–1.2) was selected to be delivered to the PA along the guide wire, and the central part of the balloon was adjusted to locate in the pulmonary annulus area. The balloon was rapidly filled with diluted contrast medium until the incisura of the balloon disappeared, after which the contrast medium was rapidly evacuated. The filling and evacuation of the balloon was repeated 2–3 times, during which the pressure was repeatedly measured.

### Statistical Analysis

Continuous variables were expressed as mean ± standard deviation, and comparisons were performed with one-way ANOVA to determine whether there were statistical differences in baseline characteristics and any prognostic indicator among the three groups. For further multiple comparisons, Tukey's multiple comparisons test was used. SPSS 24.0 software was used for statistical analysis. *P* < 0.05 was considered to indicate statistical significance.

## Results

From January 2014 to December 2017, 13 cases were enrolled into group I. The gestational weeks of prenatal PS diagnosis ranged from 22 to 36 weeks (median: 30 weeks). The average delivery gestational weeks was 39^+1^. The PG at the time of prenatal diagnosis was 57.62 ± 9.10 mmHg. However, the PG increased to 84.54 ± 17.81 mmHg around the perinatal time and reached 96.23 ± 15.67 mmHg just before the PBPV procedure. There were significant differences among the three time points (*P* < 0.05). Table 1 shows the clinical data of each individual newborn in group I. Furthermore, 11 and 18 patients were enrolled in groups PR and PO, respectively. All patients included in this study completed the 1-year follow-up with repeated echocardiographic evaluation. Three patients presented CPS with ventricular septal defect (VSD), and a set of twins showed that one fetus suffered CPS, and the other had a suspected left ventricular myocardium non-compaction; all five patients were excluded from further analysis.

**Table 1:** Characteristics of particular cases among the Int group.

	**Prenatal diagnosis of gestational weeks**	**PG at the first-time prenatal screening (mmHg)**	**Gestational week of delivery**	**PG at the time of delivery (mmHg)**	**PG before operation (mmHg)**
1	26 w	55	38^+5^ w	67	87
2	29 w	47	38^+3^ w	78	95
3	32 w	58	39^+3^ w	75	106
4	33 w	55	39^+1^ w	68	86
5	34 w	49	38^+4^ w	74	88
6	34 w	62	37^+6^ w	69	79
7	36 w	57	38^+4^ w	86	96
8	24 w	61	40^+3^ w	77	87
9	22 w	56	38^+2^ w	94	144
10	28 w	43	41^+0^ w	88	90
11	29 w	72	38^+1^ w	102	106
12	32 w	76	40^+4^ w	132	90
13	30 w	58	39^+5^ w	89	97
Average	29.92 ± 4.15 w	57.62 ± 9.10	39.10 ± 1.05 w	84.54 ± 17.81	96.23 ± 15.67

As shown in Table 2, it failed to address any significant differences for most of the parameters among the three groups, including preoperative oxygen saturation, preoperative PG, balloon/annulus ratio, and immediate PG after PBPV. However, the Int group showed the earliest time of operative age in both group PR and PO. Besides this, group PR also revealed a younger age when undergoing PBPV than group PO (*P* < 0.0001), which indicated a shorter duration of hypoxia exposure.

**Table 2:** Clinical findings before and after PBPV.

	**Group I (*n* = 13)**	**Group PR (*n* = 11)**	**Group PO (*n* = 18)**	***t1***	***t2***	***t3***
Age at PBPV (day)	9.38 ± 5.58	24.55 ± 4.87	49.11 ± 9.5	<0.0001	<0.0001	<0.0001
Preoperative oxygen saturation (%)	85.77 ± 6.96	84.82 ± 4.55	81.39 ± 6.1	0.9219	0.1273	0.3095
Hypoxic exposure time (days)	3.93 ± 3.15	22.45 ± 5.07	46.39 ± 9.65	<0.0001	<0.0001	<0.0001
Preoperative PG (mmHg)	96.23 ± 15.67	98.55 ± 11.98	101.94 ± 12.98	0.9099	0.4889	0.7926
Balloon/Pulmonary artery ratio	1.11 ± 0.16	1.13 ± 0.15	1.14 ± 0.13	0.9466	0.8165	0.9686
Immediate PG after PBPV (mmHg)	29.69 ± 15.16	29.55 ± 15.3	30.44 ± 14.57	0.9997	0.9895	0.9865
PG at 3 months after PBPV (mmHg)	20.67 ± 9.59	33.6 ± 8.55	45.43 ± 9.96	0.0054	<0.0001	0.0065
PG at 12 months after PBPV (mmHg)	21.33 ± 9.59	37.9 ± 13.07	44.71 ± 13.41	0.0058	<0.0001	0.3252
Z score for TV before PBPV	−1.95 ± 0.27	−1.84 ± 0.41	−1.79 ± 0.31	0.4378	0.1197	0.6637
Z sore for TV at 12 months after PBPV	−0.54 ± 0.47	−0.73 ± 0.36	−0.98 ± 0.19	0.2806	0.0009	0.0126

*Group I, Integrative management group; Group PR, Prenatal diagnosis group; Group PO, Postnatal diagnosis group; PG, Peak transvalvular gradient; TV, tricuspid valve; t1, Group I vs. Group PR; t2, Group I vs. Group PO; t3, Group IPR vs. Group PO*.

**Figure 1:**
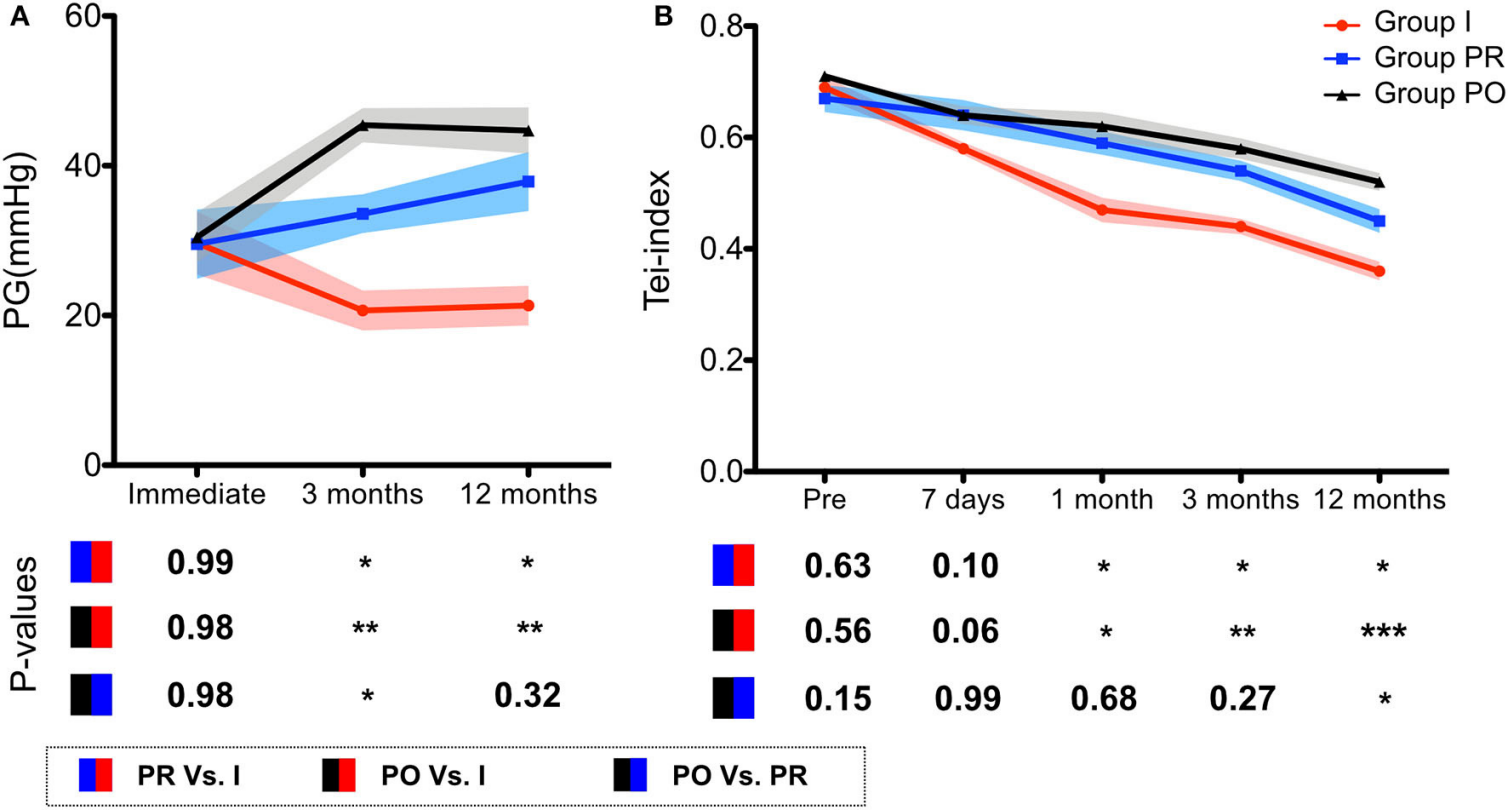
The curves demonstrate the clinical outcomes after PBPV among different groups. **(A)** The PG records from immediate, 3 and 12 months after PBPV. The results showed integrative management keeps the PG remaining around a low level, indicating an acceptable prognosis. **(B)** Tei index dropped after PBPV among all 3 groups. However, the integrative management enhanced the tendency and resulted in a better RV functional score compared to prenatal and postnatal diagnosis groups. PBPV, percutaneous balloon valvuloplasty; PG, peak transvalvular gradient; Int, integrative management group; Pre, prenatal diagnosis group; Post, postnatal diagnosis group.

PG at the 3- and 12-month follow-up in group I was lower than in both groups PR and PO after PBPV. Although PG after 3 months PBPV in group PR was lower than in group PO, there was no significant difference in this parameter measurement at the 12-month follow-up after PBPV in group PR compared to group PO cases. As shown in Figure 1A, the immediate PGs were similar among all three groups, and the PG value kept reducing after PBPV in group I and remained around 20 mmHg at the final follow-up. However, the PGs were elevated in both groups PR and PO and reached 37.9 ± 13.07 and 44.71 ± 13.41 mmHg, respectively.

The Tei index was used to evaluate RV function. Tei index of RV decreased after PBPV in coordinating with follow-up duration (7 days, 1, 3, and 12 months after PBPV, respectively) compared with the preoperative data (*P* < 0.05). In group PR, Tei index measurement at 7 days and 1 month after PBPV was dramatically reduced (*P* > 0.05). During the follow-up, it showed reduction from 3 months after PBPV (*P* < 0.05). In group PO, the Tei index gradually decreased after the 1-month follow-up (*P* < 0.05) (Figure 1B). According to the follow-up results, there was no significant difference among groups I, PR, and PO with respect to Tei index at preoperation and 7 days after PBPV (*P* > 0.05). However, the Tei index recorded in group I was lower than that in group PR and PO at each follow-up time point after PBPV (*P* < 0.05). Besides this, there was no significant difference of Tei index between groups PR and PO at 1 and 3 months after PBPV, and it was lower at the 12-month follow-up after PBPV (*P* < 0.05) (Figure 1B).

Besides this, Z score for TV was used to evaluate the capability to maintain biventricular circulation function. Before PBPV, the Z-scores of TV among the 3 groups were −1.95 ± 0.27, −1.84 ± 0.41, and −1.79 ± 0.31, respectively, which predicted biventricular heart after PBPV and presumed indication for PBPV. However, the prenatal diagnosis and perinatal integrative management demonstrated the advantages in RV functional restoration, and the Z-score of TV increased to −0.54 ± 0.47 after 1 year follow-up although the Z-score of TV in group PR was −0.73 ± 0.36. Both groups I and PR revealed an elevated Z-score compared to that of group PO of −0.98 ± 0.19.

Overall, 10 patients (one in group I, three in group PR, and six in group PO) required reintervention after PBPV within 1 year. We also found that seven patients (two and five in grouped PR and PO, respectively) had moderate pulmonary regurgitation, and four patients (one and three in groups PR and PO, respectively) showed signs of mental and psychomotor retardation during follow-up. However, there was no evidence to show significant differences among the three groups with respect to prevalence of reintervention, postoperative moderate pulmonary regurgitation, and mental and psychomotor retardation (*P* >0.05) (Table 3).

**Table 3:** Complications after operation among groups.

	**Group I (*n* = 13)**	**Group PR (*n* = 11)**	**Group PO (*n* = 18)**	***p-*value**
Reintervention (*n*)	1	3	6	0.274
Postoperative moderate pulmonary regurgitation (*n*)	0	2	5	0.1
Mental and motor retardation (*n*)	0	1	3	0.35

*Group I, Integrative management group; Group PR, Prenatal diagnosis group; Group PO, Postnatal diagnosis group*.

## Discussion

To the best of our knowledge, this is the first report that has attempted to address the impact of perinatal integrative management for CPS. Our study indicated that PBPV could be an efficient method to resolve the adverse effects of CPS as immediate PG after PBPV decreased sharply compared with preoperative status in each group. The success rate of the technique was 100% in our center without any severe adverse complications, such as threatening arrhythmia and aggressive heart dysfunction or even death. Although the oxygen saturation before PBPV among the three groups showed no difference, the hypoxia exposure time in group I was shorter in both groups PR and PO, which should be considered as a benefit from the shifted scheduled operation time for PBPV given that group I showed the earliest operative time of 9.38 ± 5.58 days. The follow-up data revealed better outcomes of PG in group I with a perfect efficiency from PBPV, and patients in groups PR and PO presented a gradual accumulation of PGs. Besides this, the Tei index also referred to better functional restoration and morphology development of RV. However, the ratio of reintervention, postoperative pulmonary valvular regurgitation, and mental and psychomotor retardation confirmed no difference during the follow-up among the three groups.

Prenatal cardiac screening has been applied in most advanced and developing countries. The medical intervention protocol was vital for screened critical congenital heart disease to obtain better outcomes during prenatal and postnatal periods (10, 17, 18). Owing to the limitation of medical technology and ethical considerations, critical CHD of the fetus is still mainly treated after birth. In theory, CPS can be treated as early as possible to provide more opportunities for right-sided development of the cardiac system and a better prognosis. Integrative perinatal management is supposed to provide the most practical clinical decision under full consideration of prenatal diagnosis, fetal and neonatal heart function and development monitoring, expected prognosis, and the capability to prepare for timely pregnancy termination following emergency intervention. Therefore, once babies with critical CHD have been delivered, they are expected to receive more timely and professional multidisciplinary intervention.

Previous studies have reported good treatment effects at immediate, short-term, and long-term evaluation results after PBPV regardless of the patient's age and valvular morphology (19, 20). PBPV is considered an efficient method for CPS. However, other studies have speculated that RV function likely originated in the fetal period and that postnatal RV growth would be restricted due to CPS. Li et al. found that there were impaired right and left ventricular mechanics and ventricular–ventricular interaction in adolescents and young adults who underwent delayed PBPV (4). Moreover, as per current treatment standards, for some patients with extremely severe PS and RV hypoplasia, the treatment can be performed during the fetal stage to restore RV development and function. The belief is that earlier relief of stenosis would result in more patient benefits (21). Accordingly, our study showed that PBPV was an efficient and safe therapeutic technology with better therapeutic effects when it was performed earlier. Once prenatal diagnosis of CPS is made, an earlier intervention can be scheduled than that in patients in group PO. Our follow-up data on PGs and Tei index supports the opinion on earlier relief of stenosis results in better outcomes based on the comparison for groups I and PR with group PO. Additionally, the integrative perinatal management revealed another step in predicting a more promising prognosis with more stable PGs and more functional RV remodeling.

In group I, the Tei index of RV was significantly decreased 7 days after PBPV, and it was closer to normal and stable with follow-up over time compared to the other two groups. In addition, the Tei index of RV also was decreased 3 months after PBPV in both groups PR and PO compared with preoperation. These results reflect that RV function showed development and functional restoration after PBPV. On the other hand, a better Tei index was recorded in group I than in groups PR and PO at 1, 3, and 12 months follow-up after PBPV, respectively. Although there was no significant difference of Tei index within 3 months after PBPV between groups PR and PO, an obvious reduction of Tei index was identified in group PR as compared to group PO, 12 months after PBPV. These data suggest that the earlier PBPV is performed, the better it is for improving RV function. Besides this, the Z-score of TV is a sensitive parameter to predict biventricular outcomes. In hypoplastic left heart syndrome (HLHS), a Z-score of TV >-2 is related to the capability to build biventricular circulation. Besides this, the Z-score of TV has also been used to demonstrate the RV development in severe pulmonary stenosis or atresia. In this study, we enrolled patients with high enough Z-scores indicating biventricular function, and we showed more efficient impacts on RV development following integrative perinatal management based on the changes of Z-score.

It is crucial to select appropriate balloons for successful PBPV to decrease implication. The balloon/annulus ratio is recommended as 1:1.2–1.4 according to the guidelines, which would result in some potential complications for small neonates such as long-term massive pulmonary regurgitation and reintervention, and some children may even need valve replacement. To avoid those complications, it is generally believed that the most appropriate balloon/valve ratio is ~1.2 times the pulmonary annular diameter, taking care to avoid ratios that are >1.3 times the pulmonary annular diameter; in addition, the younger the age, the smaller the ratio as long as it reached the clinically acceptable immediate postoperative PG (3, 22). In our study, there was no difference of balloon/annulus ratio among the three groups (group I: 1.11 ± 0.16, group PR: 1.13 ± 0.15, and group PO: 1.14 ± 0.13). We did not identify any difference between reintervention and postoperative moderate pulmonary regurgitation among the three groups even at the 1-year follow-up. Hypoxia is unavoidable for CPS, which usually leads to brain injury and affects brain development, including growth, cognition, and psychomotor functions; it causes difficulties with respect to social interaction, inattention, and emotional symptoms and impairs executive function (23, 24). The patients definitely suffered hypoxia among the three groups (preoperative oxygen saturation: 81.39–85.77%). However, the prenatal diagnosis and integrative perinatal management could help to reduce the hypoxia exposure time. Besides this, ACEI has been used among all the enrolled cases, which showed a potential benefit for RV remodeling (25, 26). Although a series of cases demonstrated that phentolamine application helps improve the severe pulmonary stenosis cases' clinical status (27), in our research, we did not provide this medication as a routine therapy, but evidence presented that phentolamine could be an alternative strategy in perinatal management (28).

## Limitations

This study has some limitations. First, the number of cases was small, which may hamper determination of a solid conclusion and statistical analysis. One of the reasons may be that the parents decided to terminate the pregnancy when CPS was identified in the fetus. Although we enrolled limited cases, we set up this preliminary analysis to demonstrate the advantages of prenatal diagnosis and perinatal integrative management. Second, this was a single-center study with only a 1-year follow-up. It is not clear at present whether the complications and mental and psychomotor retardation would vary with a longer follow-up.

## Conclusions

PBPV is an efficient and safe intervention for CPS. Prenatal diagnosis and earlier PBPV could improve the outcomes of CPS, which can be achieved by the perinatal integrated intervention strategy. Perinatal integrative management can likely enhance the advantage of prenatal diagnosis, which would shorten hypoxia exposure and benefit functional restoration of RV. We believe the integrated intervention model is feasible and an offers an important developmental direction in the treatment of critical CHD, which is not a required fetal cardiac intervention.

## Data Availability Statement

The original contributions presented in the study are included in the article/supplementary materials, further inquiries can be directed to the corresponding author/s.

## Ethics Statement

This research has been approved by the ethics committee of West China Second University Hospital, Sichuan University (2014-034). Written informed consent to participate in this study was provided by the participants' legal guardian/next of kin.

## Author Contributions

JL, GL, and YL designed this research. JL, GL, XS, CW, and HD participated in the data collection of this research. GL, JL, and YL performed the statistical analysis. KZ and YH provided supervision for this project. GL and JL wrote the original manuscript. YL revised the draft and approved the submission. All authors contributed to the article and approved the submitted version.

## Conflict of Interest

The authors declare that the research was conducted in the absence of any commercial or financial relationships that could be construed as a potential conflict of interest.
